# Is equity considered in systematic reviews of interventions for mitigating social isolation and loneliness in older adults?

**DOI:** 10.1186/s12889-022-14667-8

**Published:** 2022-12-01

**Authors:** Mohamad Tarek Madani, Leen Madani, Elizabeth Tanjong Ghogomu, Simone Dahrouge, Paul C. Hébert, Clara Juando-Prats, Kate Mulligan, Vivian Welch

**Affiliations:** 1 Bruyère Research Institute, University of Ottawa, 85 Primrose Ave, Ottawa, ON K1R 6M1 Canada; 2 Li Ka Shing Knowledge Institute, St Michael’s Hospital, Unity Health Toronto, Toronto, ON Canada; 3 Social and Behavioural Health Sciences Division, Dalla Lana School of Public Health, University of Toronto, Toronto, ON Canada

**Keywords:** Social isolation, Loneliness, Intervention, Equity, PROGRESS-plus, Overview of reviews, Systematic review, Older adults

## Abstract

**Background:**

Social isolation and loneliness affect one in four older adults in many regions around the world. Social isolation and loneliness are shown to be associated with declines in physical and mental health. Intersecting social determinants of health influence both the risk of being socially isolated and lonely as well as the access and uptake of interventions. Our objective is to evaluate what evidence is available within systematic reviews on how to mitigate inequities in access to and effectiveness of interventions.

**Methods:**

We performed an overview of reviews following methods of the *Cochrane Handbook* for Overviews of Reviews. We selected systematic reviews of effectiveness of interventions aimed at mitigating social isolation and loneliness in older adults (aged 60 or above) published in the last 10 years. In addition, we assessed all primary studies from the most recent systematic review with a broad intervention focus. We searched MEDLINE, EMBASE, PsycINFO, CINAHL, and Scopus in collaboration with a librarian scientist. We used a structured framework called PROGRESS-Plus to assess the reporting and consideration of equity. PROGRESS-Plus stands for place of residence, race/ethnicity/culture/language, occupation, gender or sex, religion, education, socioeconomic status (SES), social capital, while “plus” stands for additional factors associated with discrimination and exclusion such as age, disability, and sexual orientation. We assessed whether PROGRESS-Plus factors were reported in description of the population, examination of differential effects, or discussion of applicability or limitations.

**Results:**

We identified and assessed 17 eligible systematic reviews. We assessed all 23 primary studies from the most recent systematic review with a broad intervention focus. All systematic reviews and primary studies described the population by one or more PROGRESS-Plus factor, most commonly across place of residence and age, respectively. None of the reviews and five primary studies examined differential effects across one or more PROGRESS-Plus dimension. Nine reviews and four primary studies discussed applicability or limitations of their findings by at least one PROGRESS-Plus factor.

**Conclusions:**

Although we know that social isolation and loneliness are worse for the poorest and most socially disadvantaged older adults, the existing evidence base lacks details on how to tailor interventions for these socially disadvantaged older people.

**Supplementary Information:**

The online version contains supplementary material available at 10.1186/s12889-022-14667-8.

## Introduction

Social isolation and loneliness are global public health concerns that affect particularly older people [1]. Social isolation is the objective deficit in someone’s social network which leads to infrequent interaction with others [2]. The deficit in someone's social network may be due to retirement, death or institutionalization of friends and relatives, loss of mobility, or lack of transportation [3]. Loneliness is the subjective negative feeling that results from discrepancy or unmet needs between the desired and actual social interactions [4]. A growing body of evidence links persistent feelings of loneliness and social isolation with adverse physical and mental health problems in older adults. For instance, social isolation and loneliness are associated with increased likelihood of mortality [5]. In addition, there is strong evidence that socially isolated and lonely older adults are at an increased risk for cardiovascular diseases [6], cognitive impairment [7], dementia [8], depression [9], and anxiety [9].

Social isolation and loneliness have been on the rise during the COVID-19 pandemic due to the need to remain physically distanced, especially for older adults [10]. Therefore, tackling social isolation and loneliness has become imperative, and there is an urgent need to identify effective interventions to address this issue. Many interventions have been developed to reduce social isolation or loneliness in older adults including one-to-one, group, or mixed approaches [11]. These include diverse strategies to mitigate loneliness or social isolation such as offering activities (e.g., social or physical programs), support (e.g., discussion, counselling, therapy or education), internet training, home visiting, or service provision [12].

A scoping review of reviews proposed that a one-size-fits-all approach to addressing loneliness or social isolation in older adults does not work, given the diversity in needs of individuals and specific groups [11]. This scoping review also identified that interventions often do not target (or are not designed to reach) older adults from ethnic minorities, or from a different cultural background, or those with low socio-economic status [11]. Tailoring of interventions may be needed to address the specific challenges and meet the needs of socially disadvantaged older adults, taking into account their perspectives on social isolation and loneliness and their contexts [13, 14].

Systematic reviews are considered the gold standard for evidence to inform policy and practice [15–17]. This would make them the first resort of evidence for policy makers when looking for effective interventions to tackle social isolation and loneliness. Policy makers are interested in factors which may influence effectiveness across the population and whether interventions promote health equity [18]. Contextual factors, such as living in a low-resource setting, may result in differences across a population in terms of access to care and in unequally distributed social determinants of health which contribute to underlying inequities in health. Health inequities are defined as unnecessary and avoidable differences in health across socially stratifying factors that are unfair and unjust [19]. Lack of health equity considerations in systematic reviews has been pointed out by policy makers as a barrier to their use for decision-making [20].

In two overviews of over 50 systematic reviews on social isolation and loneliness [9, 11], it is unclear what evidence is available on how to identify and reach socially disadvantaged populations and whether interventions have the potential to mitigate health inequity.

Our objective is to evaluate what evidence is available within systematic reviews on how to mitigate inequities in access to and effectiveness of interventions which aim to reduce social isolation and loneliness in older adults.

## Methods

The methodology for this overview was conducted according to the methods of the *Cochrane Handbook* for Overview of Reviews [21] and is reported according to the reporting structure suggested by Hartling and colleagues [22] (Additional File 1: Table S1). Overviews use explicit methods to search for and identify systematic reviews related to a topic area to answer specific research questions through extracting and analyzing their results across important selected outcomes [21].

### Search strategy

After several preliminary searches which were intended to aid with identification of key words, the following databases were searched for relevant systematic reviews: MEDLINE, EMBASE, PsycINFO, CINAHL and SCOPUS. The search strategy was devised with the help of a research librarian from the University of Ottawa in MEDLINE (via OVID). The strategy was then adapted for the specific requirements of the other databases. All databases were searched from 1 Jan 2011 to 5 November 2021 to summarize the most recent available evidence. Search terms and strategies are available in Additional File 2: Tables S2-S6.

### Inclusion criteria

We included systematic reviews that assess effectiveness of interventions mitigating social isolation and/or loneliness in older adults. We defined a systematic review as systematic if it used explicit methods in the identification, selection, synthesis, and summary of primary studies [23]. These systematic reviews could be of any intervention that could alleviate loneliness and/or social isolation (e.g., physical interventions, digital interventions). Given the variability in the age range of ‘older’ populations, we adapted the cut off for older adults used by the United Nations and considered participants aged 60 years or above for inclusion [24]. We included systematic reviews with comparators against usual care, no intervention, or any other intervention. Included reviews needed to have measured loneliness and/or social isolation outcomes, either quantitatively or qualitatively. Lastly, limiters were applied in relation to language as we only included reviews written in English due to the additional resources required to translate reviews in other languages.

### Selection of reviews

All references retrieved from the search strategy were uploaded into the systematic review management software Covidence [25]. Initially, one author (MTM) performed screening at the title and abstract level to exclude references that were obviously irrelevant. Then, potentially relevant articles were retrieved and screened at full text against the inclusion criteria by one author (MTM). Single screening for selecting studies was performed instead of double screening due to lack of resources and the need to complete the project within a defined period. When the author (MTM) was uncertain whether to include or exclude an article, he consulted with another author (VW or EG). We documented all reasons for exclusion of articles at the full-text stage for entry into a PRISMA (Preferred Reporting Items for Systematic Reviews and Meta-Analyses) flowchart [26].

### Data extraction

Two authors (MTM and LM) extracted data using an extraction form which was developed in consultation with the team and an advisory committee and by pilot testing on 4 reviews. The advisory committee (SD, PH, CJP, KM) provided valuable insight as individuals with expertise working with vulnerable populations. We compared the data extracted by both authors for each article and resolved disagreements by consensus. We used the PROGRESS-Plus framework to identify dimensions across which health inequities may exist since it is recommended by the Campbell and Cochrane Equity Methods Group [27]. PROGRESS-Plus is an acronym that captures a number of socially stratifying factors understood to influence health opportunities and outcomes [28]. It represents the following dimensions: place of residence, race/ethnicity/culture/language, occupation, gender or sex, religion, education, socioeconomic status, and social capital (e.g., older adults living alone or based on marital status). The “Plus” in PROGRESS-Plus refers to any additional factors which could affect health equity such as age, disability, and sexual orientation. Definitions and examples of each PROGRESS-Plus dimension in the context of socially isolated or lonely older adults are outlined in Table 1.

**Table 1:** PROGRESS-Plus factors’ definitions and examples in the context of socially isolated or lonely older adults

PROGRESS-Plus factor	Definitions and Example
Place of residence	Residence in areas or facilities that might affect access to or quality of care ***Example:**** rural, urban, country of residence (high-, middle-, or low-income), type of residence (e.g., institutionalized, nursing home, assisted living facility), *etc
Race, ethnicity, culture, language	Differences in health outcomes exist across communities of different races, ethnicities, cultures, and languages according to region ***Example:**** white, black, Hispanic, Chinese immigrants, non-English speaking, *etc
Occupation	Occupation encompasses different situations like unsafe working environments or lack of access to employee benefits ***Example:**** employment status (e.g., unemployed), blue collar job, *etc
Gender or sex	Gender roles or sexual identities that may result in differential health risks or access to health services ***Example:**** men, women, cisgender, transgender, *etc
Religion	Religious beliefs or affiliations may limit a patient’s participation in interventions or may lead to bias and discrimination from service providers ***Example:**** Christian, Muslim, Jew, *etc
Education	Education level correlate with type of employment and income status, in addition to knowledge about health and preventative health practices ***Example:**** years of education, highest level of education completed (high-school, university, college), *etc
Socioeconomic status	Social status and income levels correlate with improvements in living conditions or access to care or preventative health practices ***Example:**** income (monthly or household), attainment or type of health insurance, *etc
Social capital	Social relationships and networks, as well as the availability of these networks to provide support ***Example:**** marital status, living alone, presence of caregiver, network size, *etc
Plus: Age	Age may be associated with independence, social capital, and health comorbidities ***Example:**** elderly, young, age range (e.g., 40–50, 70–80) *etc
Plus: Disability	Any mental health or functional impairment severe enough to reasonably believe that it might impact the ability of the individual to self-manage ***Example:**** cognitive impairment, handicap, incapacitated, chronic pain, *etc
Plus: Sexual orientation	Sexual orientation may lead to bias and discrimination from service providers ***Example:**** heterosexual, homosexual, bisexual, *etc

We collected data on eligibility for systematic reviews across intervention (e.g., types of interventions, use of screening or referrals to identify lonely or socially excluded older adults) and population characteristics. In addition, we collected data on whether interventions were designed with community or participants’ engagement. To assess that, we looked on whether participants or the community were involved in designing, developing, or delivering of the interventions. We also extracted data on whether interventions were tailored to reach socially disadvantaged populations. To determine that, we looked on whether interventions were reviewed or changed to more appropriately fit the needs and address the specific challenges of socially disadvantaged older adults.

We assessed whether the reviews’ authors considered or reported any of the PROGRESS-Plus factors when: 1) describing the characteristics of the populations of their included studies; 2) performing differential effectiveness analysis using subgroup analysis or other methods; and 3) discussing the applicability or limitations of their reviews’ findings. In addition to extracting data from systematic reviews, we decided to extract the same data from all primary studies of the most recent systematic review with a focus on broad types of interventions to assess whether primary studies describe or analyze equity-relevant data across PROGRESS-Plus dimensions. This would be an indicator to whether the availability of data within primary studies is a limitation to the description and analysis of equity within systematic reviews. The most recent systematic review with a broad intervention focus was picked because it would have the highest potential of containing overlapping primary studies used in older included systematic reviews, even if they were focused on a specific intervention type.

## Results

### Literature search

**Fig. 1:**
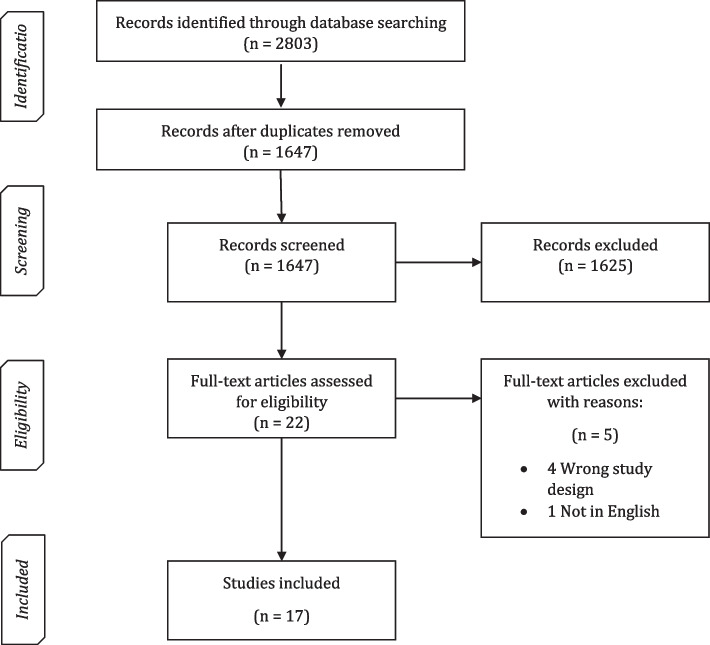
PRISMA flow chart of the selection process. In total, 2803 records were identified through searching and combining results from all databases: MEDLINE, EMBASE, CINAHL, PsycINFO, and SCOPUS. After removal of duplicates, 1647 records were screened at title and abstract level. Of these 1647 records, 22 articles were assessed at full text for eligibility. Five articles were excluded of which four had a wrong study design and one was not in English. Hence, 17 systematic reviews were included in our study for assessment

Our literature search yielded 2803 potential articles, resulting in 1647 unique articles after removal of duplicates (Fig. 1). Of the unique articles, 22 potential studies were selected for full text assessment. After applying the inclusion criteria, 17 eligible systematic reviews were included. The remaining five studies were excluded because four were not systematic reviews and one was not written in English.

### Description of included systematic reviews

We identified 17 systematic reviews of effectiveness of interventions which aim to mitigate social isolation and/or loneliness in older adults [12, 29–44]. The characteristics of these reviews are presented in Table 2. The reviews can be characterized by their intervention type: six reviews of technological interventions, one review of group reminiscence therapy interventions, one review of physical activity interventions, and nine general reviews of any intervention type. In addition, the focus of the reviews differed in which eight reviews focused on social isolation and loneliness, five reviews focused on loneliness, and four reviews focused on social isolation. Only two reviews included studies which focused on identified lonely and/or socially isolated older adults, with the remaining 15 reviews including older adults who are not necessarily lonely and/or socially isolated. Further details on the characteristics of the included systematic reviews are shown in Additional file 3: Table S7.

**Table 2:** Characteristics of the included systematic review (*n* = 17)

	**Count (n)**	**Percentage (%)**
**Review intervention type**		
Broad scope (any intervention type)	9	53
Technological interventions	6	35
Group reminiscence therapy	1	6
Physical activity	1	6
**Review focus**		
Social isolation and loneliness	8	47
Loneliness	5	29
Social isolation	4	24
**Review population characteristics**		
Older adults not necessarily lonely and/or socially isolated	15	88
Focused on lonely and/or socially isolated older adults	2	12
**Publication year**		
2019–2021	6	35
2015–2018	9	53
2011–2014	2	12

### Description of primary studies

Within the 17 systematic reviews, there were a total of 366 primary studies and a range of 6 to 39 studies per review. We assessed all primary studies from one of the 17 included systematic reviews: the most recent systematic review with a broad intervention scope [44]. This systematic review included 24 primary studies, however, we included 23 primary studies in our assessment as one study was excluded for not being written in English.

The type of interventions evaluated within these primary studies can be characterized in terms of delivery mode: 15 interventions were delivered in groups, six were delivered one-to-one, and two used a combination of one-to-one and group formats. Six studies evaluated activity interventions (e.g., social or physical programs), three evaluated internet training interventions, one evaluated home visiting, and 13 evaluated support interventions (e.g., discussion, counselling, therapy or education). As for primary studies population characteristics, 11 studies explicitly focused on identified lonely and/or socially isolated older adults, with the remaining studies including older adults who are not necessarily lonely and/or socially isolated. Further details on the characteristics as well as full citation details of the included primary studies are available in Additional file 3: Table S7.

### Description and analysis of heath equity in systematic reviews

Within the 17 systematic reviews, some of the included primary studies focused on people who were socially isolated or lonely, but none of the reviews described how these populations were identified (e.g., through case-finding or referral). In addition, none of the reviews described whether their included primary studies’ interventions were designed with community or participants’ engagement. None of the reviews also reported whether their included primary studies’ interventions were tailored to reach socially disadvantaged populations of older adults.

**Fig. 2:**
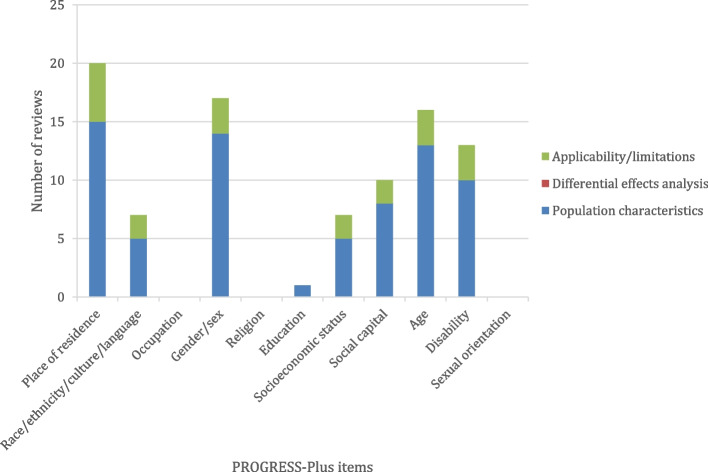
Results of the description and analysis of health equity in systematic reviews (*n* = 17). Number of reviews reporting population characteristics, differential effects analysis, and applicability or limitations across PROGRESS-Plus items. Bars do not sum to 17 because reviews were double counted if they reported multiple types of relevant data across multiple PROGRESS-Plus item

All 17 systematic reviews describe population characteristics of people included in primary studies across one or more PROGRESS-Plus item. Figure 2 shows that place of residence was the most commonly reported item (15 reviews [88%]), followed by gender or sex (14 reviews [82%]), age (13 reviews [76%]), disability (10 reviews [59%]), social capital (8 reviews [47%]), socioeconomic status and race/ethnicity/culture/language (5 reviews each [29%]), and education (1 review [6%]).

None of the 17 systematic reviews conducted analysis of differential effects using subgroup analysis or other methods across any of the PROGRESS-Plus factors (Fig. 2).

Out of the 17 systematic reviews, nine discussed applicability or limitation of interventions across one or more PROGRESS-Plus factors. Figure 2 shows that place of residence was the most commonly reported item (5 reviews [29%]), followed by gender or sex, age, and disability (3 reviews each [18%]), race/ethnicity/culture/language, socioeconomic status, and social capital (2 reviews each [12%]).

### Description and analysis of health equity in primary studies

Nine of the 23 primary studies described how older adults who are socially isolated or lonely were identified. For instance, subjects were recruited to studies through referral by health, social, church, and community agencies [45] or recruited through advertisements in local newspapers [46–48]. Two primary studies evaluated interventions designed with participants’ engagement who, for instance, were involved in planning of the content of the group meetings [47] or the development of the technology [48]. However, none of the primary studies described whether interventions were tailored to reach socially disadvantaged populations of older adults with specific needs and preferences.

**Fig. 3:**
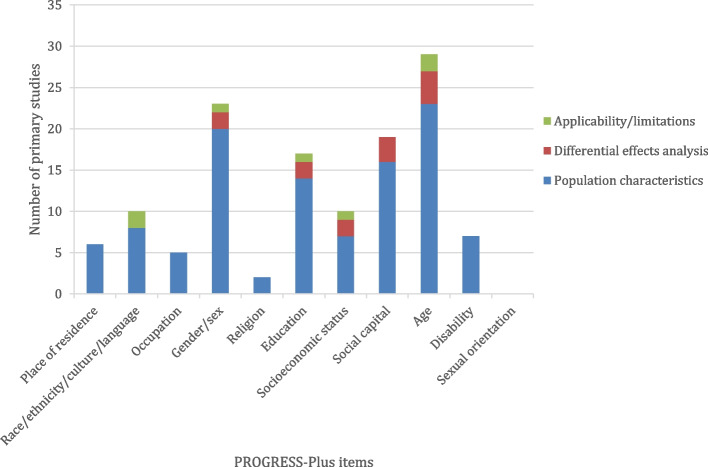
Results of the description and analysis of health equity in primary studies (*n* = 23). Number of primary studies reporting population characteristics, differential effects analysis, and applicability or limitations across PROGRESS-Plus items. Bars do not sum to 23 because studies were double counted if they reported multiple types of relevant data across multiple PROGRESS-Plus item

All 23 primary studies described population characteristics across one or more PROGRESS-Plus item. Figure 3 shows that age was the most commonly reported item (23 studies [100%]), followed by gender or sex (20 studies [87%]), social capital (16 studies [70%]), education (14 studies [61%]), race/ethnicity/culture/language (8 studies [35%]), socioeconomic status and disability (7 studies each [30%]), place of residence (6 studies [26%]), occupation (5 studies [22%]), and religion (2 studies [9%]).

Differential effectiveness across PROGRESS-Plus factors was assessed by five out of 23 (22%) primary studies. Figure 3 shows that differential effects in these studies were considered most commonly across age (4 studies [17%]), followed by social capital (3 studies [13%]), occupation, education, and socioeconomic status (2 studies each [9%]).

Out of the 23 primary studies, four discussed applicability and/or limitations across PROGRESS-Plus items. Figure 3 shows that age and race/ethnicity/culture/language were the most commonly reported items (2 studies each [9%]), followed by gender or sex, education, and socioeconomic status (1 study each [4%]).

## Discussion

We found that systematic reviews on the effectiveness of interventions to mitigate social isolation and/or loneliness in older adults did not report on differential effects nor on whether interventions are tailored to reach socially disadvantaged populations. To reduce health disparities, we need to understand what works, and what does not work for different groups. Hence, the evidence to address inequities in this field needs to be augmented.

Data on how older adults who are socially isolated and/or lonely were identified was absent from systematic reviews, although nine out of the 23 primary studies described their methods of identifying and reaching socially isolated and/or lonely older adults. Since people who are socially isolated and/or lonely are harder to reach by service providers because of their isolation or strong stigma attached to loneliness that limits the potential for individuals to ask for help, or readily reveal their needs [49], it is imperative to describe methods used to identify and reach them to ensure that people who need the intervention the most are not left out. These strategies could be helpful for policy makers when implementing interventions to mitigate the effects of social isolation and loneliness.

Community or participants engagement in intervention design has been associated with successful interventions to mitigate social isolation and loneliness [50, 51]. Enabling older adults to be involved in planning, developing, and delivering activities is most likely to lead to effective interventions [51]. This approach has the benefit of developing interventions which are better tailored to meet the needs of the people they are intended to support. Only two of the 23 assessed primary studies were designed with participant engagement, and none of the systematic reviews reported whether interventions of studies they included enabled community or participant engagement. Therefore, it is crucial for more interventions to implement input from participants or the community, and systematic review authors should report data as they become available in primary studies.

The lack of attention to health equity in these reviews is disheartening considering the importance of inequities in health for older people [52]. Only one out of the 17 systematic reviews specifically had an equity-relevant objective that aims to assess interventions that minimize social isolation and loneliness in elderly ethnic minorities [36]. Furthermore, none of the systematic reviews has explicitly considered equity when assessing the effectiveness of interventions that already target a socially disadvantaged group (socially isolated and/or lonely older adults). Even though all systematic reviews described characteristics of their primary studies populations, none of these reviews assessed differential effects. Moreover, we found that all 23 assessed primary studies reported relevant information on characteristics of their population, and five of them assessed differential intervention effects. The lack of subgroup analysis may be limited in systematic reviews due to practical limitations of the availability of data in primary studies or considerations regarding limited credibility of subgroup analyses [53].

However, barriers to performing differential effects analysis are not only restricted to availability of data. Practical limitations may prevent authors of systematic reviews to examine differential effects across pre-specified domains. Hence, evidence is often derived from post-hoc subgroup analyses, which pose problems in interpretation since such analyses are observational and not based on randomized comparisons [53]. Thus, this is challenging for researchers who are criticized by policy makers for not exploring differential effects, and criticized by statisticians for their weak methods when attempting to do so [53].

A strength of this overview of reviews is that it is the first to examine equity considerations in systematic reviews of effectiveness of interventions that mitigate social isolation and/or loneliness in older adults. Also, a systematic search of the literature was performed which encompassed various databases and included a comprehensive search strategy.

Some of the limitations in this overview include that only one author selected reviews for feasibility reasons. Since the search for reviews was limited to those written in English, it is possible that potentially relevant reviews were omitted. Limiting reviews to those published in the last 10 years could also have omitted relevant reviews, however, this decision was made to assess the recent literature to provide a better understanding of the state of the current knowledge. Because we are assessing how these reviews evaluated equity, it is unlikely that the conclusion will change by missing some reviews. We also assessed all primary studies from one included systematic review and not all systematic reviews, therefore our findings from primary studies are not fully representative of all primary studies on this question. This analysis was done as an indicator for the availability of equity-relevant data within primary studies.

## Conclusion

Although social isolation and loneliness are worse for the poorest and most socially disadvantaged populations [54], the existing evidence base of systematic reviews of effectiveness of interventions mitigating social isolation and loneliness in older adults lacks equity-relevant data. Even though sociodemographic data of the interventions’ population were collected in all systematic reviews, they were not used for quality improvement or assessing differential effects across equity factors. Our results justify the need to encourage greater consideration of equity in systematic reviews. Without greater emphasis on equity considerations, it is possible that efforts to improve health for the socially disadvantaged populations may go to waste, or worse, contribute inadvertently to widening inequities in health access, health distribution, and health outcomes [55]. Therefore, it is imperative to understand whether those who need the interventions the most are identified and reached, and whether interventions are tailored to meet their needs and are effective in improving their health outcomes.

There is a need to continue promoting the analysis of equity considerations in both primary studies and systematic reviews in this field. This requires that equity factors such as PROGRESS-Plus are included as part of learning health systems from program proposal to design, data collection, evaluation, and decision-making. Community inclusion is a fundamental component that should be considered. We urge attention and support to this shift from funders, ethics boards, researchers, decision-makers, clinicians/providers, service users, communities, and social scientists.

## Supplementary Information

**Additional file 1.** Preferred Reporting Items for Systematic Reviews and Meta-analyses (PRISMA) and their applicability to overviews of reviews.

**Additional file 2.** Search terms and strategy.

**Additional file 3.** Tables showing further details on characteristics of the included systematic reviews and primary studies.

## Data Availability

All data generated or analyzed during this study are included in this published article and its supplementary information files.

## Abbreviations

PROGRESS: Place of residence, Race/ethnicity/culture/language, Occupation, Gender or sex, Religion, Education, Socioeconomic status, Social capital and network
PRISMA: Preferred Reporting Items for Systematic Reviews and Meta-Analyses
