# Supplementary material for: Is equity considered in systematic reviews of interventions for mitigating social isolation and loneliness in older adults?
Source: BMC Public Health. 2022 Dec 1;22:2241. doi: 10.1186/s12889-022-14667-8 (PMC9713122; doi:10.1186/s12889-022-14667-8)
Supplement: Supplementary file 2 — Additional file 2. Search terms and strategy. [file 12889_2022_14667_MOESM2_ESM.docx]

**Is equity considered in systematic reviews of interventions for mitigating social isolation and loneliness in older adults?**

Mohamad Tarek Madani^1^, Leen Madani^1^, Elizabeth Tanjong Ghogomu^1^, Simone Dahrouge^1^, Paul C. Hébert^1^, Clara Juando-Prats^2^, Kate Mulligan^3^, and Vivian Welch^1^*

^1^Bruyère Research Institute, University of Ottawa, 85 Primrose Ave,

Ottawa, ON K1R 6M1, Canada; ^2^Li Ka Shing Knowledge Institute, St Michael's Hospital, Unity Health Toronto, Toronto, ON, Canada; ^3^Social and Behavioural Health Sciences Division, Dalla Lana School of Public Health, University of Toronto, Toronto, ON, Canada

***Corresponding author**: Vivian Welch

**Email**: [vwelch@campbellcollaboration.org](mailto:vwelch@campbellcollaboration.org)

**Keywords:** Social isolation, Loneliness, Intervention, Equity, PROGRESS-Plus, Overview of reviews, Systematic review, Older adults

**Table S2**. **MEDLINE** **search strategy.** The search terms for MEDLINE are listed above, along with the number of hits obtained from searching each query. The total number of hits obtained from searching MEDLINE was 459. This search strategy was adapted for the other electronic databases: EMBASE, PsycINFO, CINAHL, and SCOPUS.

| **#** | **Query** | **Results** |
| --- | --- | --- |
| 1 | loneliness/ | 4850 |
| 2 | social isolation/ | 15175 |
| 3 | lonel*.ti,ab,kf. | 9734 |
| 4 | (social* adj3 (isolat* or exclu* or contact* or support* or network* or relation*)).ti,ab,kf. | 105630 |
| 5 | 1 or 2 or 3 or 4 | 122752 |
| 6 | exp aged/ | 3327241 |
| 7 | (old* adj3 (person* or adult* or people or patient*)).ti,ab,kf. | 320320 |
| 8 | (elder* or geriatric* or senior* or retire*).ti,ab,kf. | 394455 |
| 9 | 6 or 7 or 8 | 3611758 |
| 10 | Review Literature as Topic/ or (systematic adj2 review*).mp. or meta-analysis/ or meta-analysis.mp. | 373058 |
| 11 | 5 and 9 and 10 | 539 |
| 12 | limit 11 to yr="2011 -Current" | 459 |

**Table S3**. **EMBASE** **search strategy.** The search terms for EMBASE are listed above, along with the number of hits obtained from searching each query. The total number of hits obtained from searching EMBASE was 1102.

| **#** | **Query** | **Results** |
| --- | --- | --- |
| 1 | loneliness/ | 10892 |
| 2 | exp social isolation/ | 29097 |
| 3 | lonel*.ti,ab,kf. | 12089 |
| 4 | (social* adj3 (isolat* or exclu* or contact* or support* or network* or relation*)).ti,ab,kf. | 132113 |
| 5 | 1 or 2 or 3 or 4 | 159927 |
| 6 | exp aged/ | 3435435 |
| 7 | (old* adj3 (person* or adult* or people or patient*)).ti,ab,kf. | 470891 |
| 8 | (elder* or geriatric* or senior* or retire*).ti,ab,kf. | 560379 |
| 9 | 6 or 7 or 8 | 3855754 |
| 10 | exp Review/ or (systematic adj2 review*).mp. or meta-analysis/ or meta-analysis.mp. | 3088094 |
| 11 | 5 and 9 and 10 | 2076 |
| 12 | limit 11 to yr="2011 -Current" | 1102 |

**Table S4**. **PsycINFO search strategy.** The search terms for PsycINFO are listed above, along with the number of hits obtained from searching each query. The total number of hits obtained from searching PsycINFO was 159.

| **#** | **Query** | **Results** |
| --- | --- | --- |
| 1 | loneliness/ | 5255 |
| 2 | social isolation/ | 7973 |
| 3 | lonel*.ti,ab. | 12215 |
| 4 | (social* adj3 (isolat* or exclu* or contact* or support* or network* or relation*)).ti,ab. | 138176 |
| 5 | 1 or 2 or 3 or 4 | 151040 |
| 6 | older adulthood/ | 8222 |
| 7 | (old* adj3 (person* or adult* or people or patient*)).ti,ab. | 98753 |
| 8 | (elder* or geriatric* or senior* or retire*).ti,ab. | 118320 |
| 9 | 6 or 7 or 8 | 197563 |
| 10 | exp Literature review/ or (systematic adj2 review*).mp. or meta-analysis/ or meta-analysis.mp. | 87565 |
| 11 | 5 and 9 and 10 | 234 |
| 12 | limit 11 to yr="2011 -Current" | 159 |

**Table S5**. **CINAHL search strategy.** The search terms for CINAHL are listed above, along with the number of hits obtained from searching each query. The total number of hits obtained from searching CINAHL was 299.

| **#** | **Query** | **Results** |
| --- | --- | --- |
| S11 | S5 and S9 and S10 Limiters - Published Date: 20110101-20211231 | 299 |
| S10 | TI ( (Systematic N2 review*) or meta-analysis ) OR AB ( (Systematic N2 review*) or meta-analysis ) | 158,010 |
| S9 | S6 or S7 or S8 | 990,809 |
| S8 | TI ( (elder* or geriatric* or senior* or retire*) ) OR AB ( (elder* or geriatric* or senior* or retire*) ) | 163,962 |
| S7 | TI ( (old* N3 (person* or adult* or people or patient*)) ) OR AB ( (old* N3 (person* or adult* or people or patient*)) ) | 141, 738 |
| S6 | (MH “Aged+”) | 889,414 |
| S5 | S1 or S2 or S3 or S4 | 77,518 |
| S4 | TI ( (social* N3 (isolat* or exclu* or contact* or support* or network* or relation*) ) OR AB ( (social* N3 (isolat* or exclu* or contact* or support* or network* or relation*) ) | 65,898 |
| S3 | TI lonel* OR AB lonel* | 6,527 |
| S2 | (MH "Social Isolation") | 9,581 |
| S1 | (MH "Loneliness")Expanders | 5,137 |

**Table S6**. **SCOPUS search strategy.** The search terms for SCOPUS are listed above, along with the number of hits obtained from searching each query. The total number of hits obtained from searching SCOPUS was 784.

| **#** | **Query** | **Results** |
| --- | --- | --- |
| 11 | ( ( TITLE-ABS-KEY ( ( social* W/3 ( isolat* OR exclu* OR contact* OR support* OR network* OR relation* ) ) ) ) OR ( TITLE-ABS-KEY ( ( lonel* ) ) ) ) AND ( ( TITLE-ABS-KEY ( ( old* W/3 ( person* OR adult* OR people OR patient* OR aged* ) ) ) ) OR ( TITLE-ABS-KEY ( ( elder* OR geriatric* OR senior* OR retire* ) ) ) ) AND ( ( TITLE-ABS-KEY ( ( systematic W/2 review* ) ) ) OR ( TITLE-ABS-KEY ( ( meta-analysis ) ) ) ) AND PUBYEAR > 2010 | 784 |
| 10 | ( ( TITLE-ABS-KEY ( ( social* W/3 ( isolat* OR exclu* OR contact* OR support* OR network* OR relation* ) ) ) ) OR ( TITLE-ABS-KEY ( ( lonel* ) ) ) ) AND ( ( TITLE-ABS-KEY ( ( old* W/3 ( person* OR adult* OR people OR patient* OR aged* ) ) ) ) OR ( TITLE-ABS-KEY ( ( elder* OR geriatric* OR senior* OR retire* ) ) ) ) AND ( ( TITLE-ABS-KEY ( ( systematic W/2 review* ) ) ) OR ( TITLE-ABS-KEY ( ( meta-analysis ) ) ) ) | 900 |
| 9 | ( TITLE-ABS-KEY ( ( systematic W/2 review* ) ) ) OR ( TITLE-ABS-KEY ( ( meta-analysis ) ) ) | 541,440 |
| 8 | TITLE-ABS-KEY ( ( meta-analysis ) ) | 331,333 |
| 7 | TITLE-ABS-KEY ( ( systematic W/2 review* ) ) | 380,743 |
| 6 | ( TITLE-ABS-KEY ( ( old* W/3 ( person* OR adult* OR people OR patient* OR aged* ) ) ) ) OR ( TITLE-ABS-KEY ( ( elder* OR geriatric* OR senior* OR retire* ) ) ) | 1,416,519 |
| 5 | TITLE-ABS-KEY ( ( elder* OR geriatric* OR senior* OR retire* ) ) | 1,070,579 |
| 4 | TITLE-ABS-KEY ( ( old* W/3 ( person* OR adult* OR people OR patient* OR aged* ) ) ) | 497,421 |
| 3 | ( TITLE-ABS-KEY ( ( social* W/3 ( isolat* OR exclu* OR contact* OR support* OR network* OR relation* ) ) ) ) OR ( TITLE-ABS-KEY ( ( lonel* ) ) ) | 502,666 |
| 2 | TITLE-ABS-KEY ( ( lonel* ) ) | 21,680 |
| 1 | TITLE-ABS-KEY ( ( social* W/3 ( isolat* OR exclu* OR contact* OR support* OR network* OR relation* ) ) ) | 487,951 |
