# Supplementary material for: Is equity considered in systematic reviews of interventions for mitigating social isolation and loneliness in older adults?
Source: BMC Public Health. 2022 Dec 1;22:2241. doi: 10.1186/s12889-022-14667-8 (PMC9713122; doi:10.1186/s12889-022-14667-8)
Supplement: Supplementary file 3 — Additional file 3. Tables showing further details on characteristics of the included systematic reviews and primary studies. [file 12889_2022_14667_MOESM3_ESM.docx]

**Is equity considered in systematic reviews of interventions for mitigating social isolation and loneliness in older adults?**

Mohamad Tarek Madani^1^, Leen Madani^1^, Elizabeth Tanjong Ghogomu^1^, Simone Dahrouge^1^, Paul C. Hébert^1^, Clara Juando-Prats^2^, Kate Mulligan^3^, and Vivian Welch^1^*

^1^Bruyère Research Institute, University of Ottawa, 85 Primrose Ave,

Ottawa, ON K1R 6M1, Canada; ^2^Li Ka Shing Knowledge Institute, St Michael's Hospital, Unity Health Toronto, Toronto, ON, Canada; ^3^Social and Behavioural Health Sciences Division, Dalla Lana School of Public Health, University of Toronto, Toronto, ON, Canada

***Corresponding author**: Vivian Welch

**Email**: [vwelch@campbellcollaboration.org](mailto:vwelch@campbellcollaboration.org)

**Keywords:** Social isolation, Loneliness, Intervention, Equity, PROGRESS-Plus, Overview of reviews, Systematic review, Older adults

**Table S7**. **Characteristics of included systematic reviews on effectiveness of interventions to mitigate social isolation and/or loneliness in older adults (n = 17).** References used correspond to the respective reference number of each systematic review in the original manuscript.

| **Author(s), year of publication, (ref)** | **Aims and objectives of review** | **Intervention type e.g., broad, or specific** | **Focus e.g., social isolation, loneliness, or both** | **Number of included primary studies** |
| --- | --- | --- | --- | --- |
| Dickens et al., 2011 (12) | The aim of this review was to assess the effectiveness of interventions designed to alleviate social isolation and loneliness in older people. | Broad | Social isolation and loneliness | 32 |
| Choi et al., 2012 (29) | This study conducted a meta-analysis to examine the effectiveness of computer and Internet training interventions intended to reduce loneliness and depression in older adults. | Specific: computer and internet training interventions | Loneliness | 6 |
| Cohen-Mansfield and Perach, 2015 (30) | The purpose of the present study is threefold: (1) to review the utility of interventions for loneliness for older adults in studies published between the years 1996 and 2011 and pinpoint their strengths and weaknesses; (2) to identify which interventions are efficacious for which specific subpopulations; and (3) to clarify what knowledge relating to loneliness interventions is lacking. | Broad | Loneliness | 28 |
| Syed Elias et al., 2015 (31) | The aim of this paper was to systematically review the literature in order to explore the effectiveness of group reminiscence therapy for older adults with loneliness, anxiety and depression in long-term care. | Specific: group reminiscence therapy | Loneliness | 8 |
| Franck et al., 2015 (32) | This systematic review update focuses particularly on studies from the 5 years since the previous similar review to answer the question: What effective interventions exist that address social isolation and depression in aged care clients living in rural settings? | Broad | Social isolation | 6 |
| Chen and Schulz, 2016  (33) | This systematic review explored the effects of ICT interventions on reducing social isolation of the elderly. | Specific: information communication technology interventions | Social isolation | 25 |
| Gardiner et al., 2016 (34) | The aim of this study was to conduct an integrative review to identify the range and scope of interventions that target social isolation and loneliness among older people, to gain insight into why interventions are successful and to determine the effectiveness of those interventions. | Broad | Social isolation and loneliness | 39 |
| Khosravi et al., 2016 (35) | This study undertakes a systematic literature review of empirical studies on various types of technologies and their effectiveness in alleviating social isolation among seniors. | Specific: technological interventions | Social isolation | 34 |
| Pool et al., 2017 (36) | The goal of this article was to identify effective interventions that improve social participation, and minimise social isolation and loneliness in community dwelling elderly ethnic minorities. | Broad | Social isolation and loneliness | 6 |
| Poscia et al., 2018 (37) | This systematic review aims to summarize and update the current knowledge on the effectiveness of the existing interventions for alleviating loneliness and social isolation among older persons. | Broad | Social isolation and loneliness | 20 |
| Shvedko et al., 2018 (38) | This article reviews the effects of physical activity (PA) interventions on social isolation, loneliness or low social support in older adults | Specific: physical activity interventions | Social isolation and loneliness | 38 |
| Quan et al., 2020 (39) | This study aimed to review loneliness interventions for older adults living in long-term care (LTC) facilities over the past 10 years, to categorize interventions by type, and to compare effectiveness of loneliness interventions in these settings. | Broad | Loneliness | 15 |
| Choi and Lee, 2021 (40) | To investigate the development trends of and summarize the effects of ICT interventions designed for the elderly to reduce social isolation and loneliness. | Specific: Information communication technology interventions | Social isolation and loneliness | 23 |
| Heins et al., 2021 (41) | This systematic review aims to provide a systematic overview of the effects of technological interventions that target social participation in community-dwelling older adults with and without dementia. | Specific: technological interventions | Social isolation and loneliness | 36 |
| Manjunath et al., 2021 (42) | We completed a systematic review evaluating the effectiveness of 20 interventions used to combat social isolation in older adults | Broad | Social isolation | 20 |
| Shah et al., 2021 (43) | The objective of this study is to assess the effectiveness of DTIs in reducing loneliness in older adults. | Specific: digital technology interventions | Loneliness | 6 |
| Tong et al., 2021 (44) | Therefore, this systematic review was designed to summarize and update the current knowledge about the efficacy of existing interventions for alleviating social isolation and loneliness among older adults. | Broad | Social isolation and loneliness | 24 |

**Table S8**. **Characteristic of included primary studies from the most recent systematic review with a focus on broad types of interventions (n = 23).** References used correspond to the respective reference number of each primary study in the supporting references below in the Supporting information.

| **Author(s), year of publication, country (ref)** | **Aims and objectives of primary study** | **Participants** | **Delivery mode** | **Intervention type** |
| --- | --- | --- | --- | --- |
| Harris and Bodden, 1978, USA (1) | The present investigation is an experimental attempt to (a) evaluate the two main competing theories of effective psychological adjustment to aging and (b) shed some light on the question of what types of counseling intervention might prove effective in facilitating the psychological well-being of the aged. | Community-dwelling, Meals on Wheels recipients | Group activity | Social activity |
| Constantino, 1988, USA (2) | The present experimental study was designed to test the efficacy of two group interventions - a bereavement crisis intervention and a social adjustment intervention - in reducing depression and increasing socialization among widows. | Community-dwelling, widows | Group activity | Support group |
| Lökk, 1990, Sweden (3) | The purpose of the present study was to evaluate the psychological effects of a controlled intervention programme aiming at enhanced personal control, social activation, autonomy, and life satisfaction. | Community-dwelling, handicapped people | Group activity | Discussion group |
| Heller et al., 1991, USA (4) | The goal of the current project were to determine whether indigenous peer telephone dyads could be established among low-income elderly women and whether such supportive contacts were effective in maintaining and enhancing health, activity, and morale. | Low-income housing residents | One-to-one | Telephone support |
| Brennan et al., 1995, USA (5) | The purpose of the study reported here was to examine the effects of a computer network. | Community-dwelling, Caregivers of Alzheimer's Disease (AD) sufferers | One-to-one | Computer support network |
| Morrow-Howell et al., 1998, USA (6) | This article presents an evaluation of Link-Plus, a social work service provided over the telephone under the auspices of a suicide prevention hot line agency. | Elderly at increased risk of suicide | One-to-one | Telephone support |
| MacIntyre et al., 1999, Canada (7) | The purpose of our study was to evaluate this volunteer community programme by determining the effectiveness of a friendly visitor in the volunteer programme. | Recipients of home nursing & homemaking services | One-to-one | Home visiting |
| White et al., 2002, USA (8) | This randomized controlled trial assessed the psychosocial impact of providing Internet access to older adults over a five-month period. | Nursing home and congregate housing residents | Group activity | Internet training |
| Fukui et al., 2003, Japan (9) | To examine the effects of a psychosocial group intervention on loneliness and social support in Japanese women with breast cancer. | Women with primary breast cancer | Group activity | Education/support group |
| Savelkoul and de Wittle, 2004, Netherlands (10) | To investigate in a randomized controlled trial the effects of mutual support groups in rheumatic diseases on social network size, social skills, loneliness, daily functioning, and life satisfaction as well as to identify patients’ perceptions of the support group. | Chronic rheumatic diseases patients | Group activity | Coping education group |
| Kremers et al., 2006, Netherlands (11) | In the present randomized controlled trial (RCT) it was investigated whether single women, 55 years of age and older, improved with regard to self-management ability, well-being, and social and emotional loneliness after having participated in a newly designed self-management group intervention based on the Self-Management of Well-being (SMW) theory. | Community-dwelling, single women | Group activity | Self-management group |
| Drentea et al., 2006, USA (12) | We examine the extent to which an intervention that helps spouse-caregivers mobilize their social support network, helps them better adapt to the caregiving role. | Caregivers to Alzheimer's disease (AD) sufferers | One-to-one and group | Counselling/support group |
| Ollonqvist et al., 2008, Finland (13) | The primary objective of the present report was to assess whether a network-based rehabilitation programme has an effect on emotional loneliness among community-dwelling older people at high risk of long-term institutional care. | Community-dwelling, frail, at risk of institutionalisation within 2 years due to progressively decreasing functional capacity | Group activity | Physical activity |
| Slegers et al., 2008, Netherlands (14) | We carried out a randomized, controlled intervention study that aimed to examine the causal relationship between computer use and measures of physical well-being, social well-being, emotional well-being, development and activity, and autonomy. | Community-dwelling, no prior computer experience | One-to-one | Computer/internet training |
| Routasalo et al., 2009, Finland (15) | This paper is a report of a study to explore the effects of psychosocial group nursing intervention on older people’s feelings of loneliness, social activity and psychological well-being. | Community-dwelling, reported feelings of loneliness | Group activity | Social activity |
| Bøen et al., 2012, Norway (16) | The objectives were to examine the effect of a preventive senior centre group programme consisting of weekly meetings, on social support, depression and quality of life. | Elders within senior centres | Group activity | Physical activity/Discussion groups |
| Saito et al., 2012, Japan (17) | We developed an intervention program that aimed to prevent social isolation by improving community knowledge and networking with other participants and community ‘‘gatekeepers’’, and as a result, lead to well-being among older Japanese migrants. | Elderly who relocated to different city within 2 years | Group activity | Educational, cognitive, and social support program |
| Black et al., 2014, USA (18) | Given evidence that chronic stress leads to a progressive rise in inflammation in older adults, and that both stress and increase expression of genes bearing NF-κB response elements are associated with loneliness, we hypothesized that TCC would reduce stress and slow the rate of increase in NF-κB levels in lonely older adults, as compared to those who receive a stress and health education (SHE) intervention. | Lonely, naive to Tai Chi | Group activity | Behavioural therapy (meditation/physical activity) |
| Mountain et al., 2014, UK (19) | We undertook a parallel-group randomised controlled trial to evaluate the effectiveness and cost effectiveness of telephone befriending for the maintenance of HRQoL in older people. | Living independently, with good coginitive function | One-to-one and group | Telephone support |
| Chan et al., 2017, Hong Kong (20) | To test the feasibility and preliminary effectiveness of a tai chi qigong program with the assistance of elderly neighborhood volunteers in strengthening social networks and enhancing the psychosocial well-being of hidden elderly. | Community-dwelling, did not engage in any social activities | Group activity | Behavioural therapy (meditation/physical activity) |
| Czaja et al., 2017, USA (21) | Information and communication technology holds promise in terms of providing support and reducing isolation among older adults. We evaluated the impact of a specially designed computer system for older adults, the Personal Reminder Information and Social Management (PRISM) system. | Living alone in independent housing | One-to-one | Computer training |
| Lai et al., 2020, Canada (22) | We examined the effectiveness of a peerbased intervention in reducing loneliness, social isolation, and improving psychosocial well-being with a sample of aging Chinese immigrants. | Community-dwelling older Chinese immigrants | Group activity | Peer support services |
| Ristolainen et al., 2020, Finland (23) | This study examines the effects of ‘participatory group-based care management’ conducted among community-dwelling older adults living alone in Central and Eastern Finland. The intervention aimed to promote wellbeing and quality of life (QoL) using a needs-based and participatory approach. | Older adults living alone | Group activity | Social support, counselling, and activities |

**Supporting references**

1. Harris, J. E., and Bodden, J. L. (1978) An activity group experience for disengaged elderly persons. *Journal of Counseling Psychology*. **25**, 325–330

2. Constantino, R. E. (1988) Comparison of two group interventions for the bereaved. *Image J Nurs Sch*. **20**, 83–87

3. Lökk, J. (1990) Emotional and social effects of a controlled intervention study in a day-care unit for elderly patients. *Scand J Prim Health Care*. **8**, 165–172

4. Heller, K., Thompson, M. G., Trueba, P. E., Hogg, J. R., and Vlachos-Weber, I. (1991) Peer Support Telephone Dyads for Elderly Women: Was This the Wrong Intervention? *American Journal of Community Psychology*. **19**, 53–74

5. Brennan, P. F., Moore, S. M., and Smyth, K. A. (1995) The effects of a special computer network on caregivers of persons with Alzheimer’s disease. *Nurs Res*. **44**, 166–172

6. Morrow-Howell, N., Becker-Kemppainen, S., and Judy, L. (1998) Evaluating an Intervention for the Elderly at Increased Risk of Suicide. *Research on Social Work Practice*. **8**, 28–46

7. MacIntyre, I., Corradetti, P., Roberts, J., Browne, G., Watt, S., and Lane, A. (1999) Pilot study of a visitor volunteer programme for community elderly people receiving home health care. *Health Soc Care Community*. **7**, 225–232

8. White, H., McConnell, E., Clipp, E., Branch, L. G., Sloane, R., Pieper, C., and Box, T. L. (2002) A randomized controlled trial of the psychosocial impact of providing internet training and access to older adults. *Aging Ment Health*. **6**, 213–221

9. Fukui, S., Koike, M., Ooba, A., and Uchitomi, Y. (2003) The effect of a psychosocial group intervention on loneliness and social support for Japanese women with primary breast cancer. *Oncol Nurs Forum*. **30**, 823–830

10. Savelkoul, M., and de Witte, L. P. (2004) Mutual support groups in rheumatic diseases: Effects and participants’ perceptions. *Arthritis Rheum*. **51**, 605–608

11. Kremers, I. P., Steverink, N., Albersnagel, F. A., and Slaets, J. P. J. (2006) Improved self-management ability and well-being in older women after a short group intervention. *Aging Ment Health*. **10**, 476–484

12. Drentea, P., Clay, O. J., Roth, D. L., and Mittelman, M. S. (2006) Predictors of improvement in social support: Five-year effects of a structured intervention for caregivers of spouses with Alzheimer’s disease. *Soc Sci Med*. **63**, 957–967

13. Ollonqvist, K., Palkeinen, H., Aaltonen, T., Pohjolainen, T., Puukka, P., Hinkka, K., and Pöntinen, S. (2008) Alleviating Loneliness among Frail Older People – Findings from a Randomised Controlled Trial. *International Journal of Mental Health Promotion*. **10**, 26–34

14. Slegers, K., van Boxtel, M. P. J., and Jolles, J. (2008) Effects of computer training and Internet usage on the well-being and quality of life of older adults: a randomized, controlled study. *J Gerontol B Psychol Sci Soc Sci*. **63**, P176-184

15. Routasalo, P. E., Tilvis, R. S., Kautiainen, H., and Pitkala, K. H. (2009) Effects of psychosocial group rehabilitation on social functioning, loneliness and well-being of lonely, older people: randomized controlled trial. *J Adv Nurs*. **65**, 297–305

16. Bøen, H., Dalgard, O. S., Johansen, R., and Nord, E. (2012) A randomized controlled trial of a senior centre group programme for increasing social support and preventing depression in elderly people living at home in Norway. *BMC Geriatr*. **12**, 20

17. Saito, T., Kai, I., and Takizawa, A. (2012) Effects of a program to prevent social isolation on loneliness, depression, and subjective well-being of older adults: a randomized trial among older migrants in Japan. *Arch Gerontol Geriatr*. **55**, 539–547

18. Black, D. S., Irwin, M. R., Olmstead, R., Ji, E., Crabb Breen, E., and Motivala, S. J. (2014) Tai chi meditation effects on nuclear factor-κB signaling in lonely older adults: a randomized controlled trial. *Psychother Psychosom*. **83**, 315–317

19. Mountain, G. A., Hind, D., Gossage-Worrall, R., Walters, S. J., Duncan, R., Newbould, L., Rex, S., Jones, C., Bowling, A., Cattan, M., Cairns, A., Cooper, C., Edwards, R. T., and Goyder, E. C. (2014) “Putting Life in Years” (PLINY) telephone friendship groups research study: pilot randomised controlled trial. *Trials*. **15**, 141

20. Chan, A. W., Yu, D. S., and Choi, K. C. (2017) Effects of tai chi qigong on psychosocial well-being among hidden elderly, using elderly neighborhood volunteer approach: a pilot randomized controlled trial. *Clin Interv Aging*. **12**, 85–96

21. Czaja, S. J., Boot, W. R., Charness, N., Rogers, W. A., and Sharit, J. (2018) Improving Social Support for Older Adults Through Technology: Findings From the PRISM Randomized Controlled Trial. *Gerontologist*. **58**, 467–477

22. Lai, D. W. L., Li, J., Ou, X., and Li, C. Y. P. (2020) Effectiveness of a peer-based intervention on loneliness and social isolation of older Chinese immigrants in Canada: a randomized controlled trial. *BMC Geriatr*. **20**, 356

23. Ristolainen, H., Kannasoja, S., Tiilikainen, E., Hakala, M., Närhi, K., and Rissanen, S. (2020) Effects of “participatory group-based care management” on wellbeing of older people living alone: a randomized controlled trial. *Arch Gerontol Geriatr*. **89**, 104095
